# Construction and validation of an Entrepreneurship Measurement Instrument for nursing students

**DOI:** 10.17533/udea.iee.v42n1e12

**Published:** 2024-04-29

**Authors:** Isabel del Arco Bravo, Mercé Muñoz Gimeno

**Affiliations:** 1 Pedagogue and Psychopedagogue, Ph.D. Full Profesor, Facultat de Educación, Psicología y Trabajo Social. Universidad de Lleida. Lleida, Spain. Email: Isabel.delarco@udl.cat. (Corresponding author) Universitat de Lleida Facultat de Educación Psicología y Trabajo Social Universidad de Lleida Lleida Spain Isabel.delarco@udl.cat; 2 Nurse, Ph.D. Professor, Escuela Universitaria de Enfermería y Fisioterapia Gibernau (Centro adscrito a Universidad Autónoma de Barcelona). Barcelona, Spain. Email: mercemunozg@gmail.com Universitat Autónoma de Barcelona Escuela Universitaria de Enfermería y Fisioterapia Gibernau Universidad Autónoma de Barcelona Barcelona Spain mercemunozg@gmail.com

**Keywords:** entrepreneurship, validation study, psychometrics, competency-based education, students, nursing., emprendimiento, estudio de validación, psicometría, educación basada en competencia, estudiantes de enfermería., empreendedorismo, estudo de validação, psicometria, ensino baseado em competências, ensino baseado em competências, estudantes de enfermagem.

## Abstract

**Objective.:**

To develop a valid and reliable scale to measure entrepreneurship competences of nursing students, by assessing the level of development of diverse entrepreneurship dimensions.

**Methods.:**

An Entrepreneurship Measurement Instrument, Catalonia (IME.Cat) was constructed, by adapting two existing instruments, and a psychometric study was performed to address the validity of the content and the construct, and the reliability. The internal consistency and the discrimination capacity of the instrument’s items were examined.

**Results.:**

The IME.Cat scale showed a high reliability (α=0.89) for the complete set of items. The Cronbach’s α value of the individual dimensions were: Problem management=0.78; Creativity=0.76; Personal confidence =0.64; and Risk acceptance =0.46. The corrected homogeneity indices for each of the item in the instrument were high (>0.40). The Confirmatory Factorial Analysis validated the proposed structure of the items according to dimension.

**Conclusion.:**

The IME.Cat scale showed solid psychometric values for assessing the entrepreneurship competences of nursing students within its dimensions, which are fundamental for the professional development of nursing.

## Resumen

**Objetivo.:**

Desarrollar una escala válida y fiable para medir competencias emprendedoras para estudiantes de enfermería, evaluando el nivel de desarrollo en diversas dimensiones del emprendimiento.

**Métodos.:**

Se construyó el Instrumento de Medición Emprendedora Cataluña (IME.Cat) adaptando dos instrumentos existentes y se llevó a cabo un estudio psicométrico que abordó la validez de contenido, de constructo y la fiabilidad. Se examinaron la consistencia interna y la capacidad de discriminación de los ítems del instrumento.

**Resultados.:**

La escala IME.Cat mostró una alta fiabilidad (α=0.89) para el conjunto completo de 25 ítems. Los valores del α de Cronbach de las dimensiones individuales fueron: Manejo de problemas=0.78; Creatividad=0.76; Seguridad personal=0.64; y Aceptación del riesgo=0.46. Los índices de homogeneidad corregidos para cada ítem del instrumento fueron elevados (>0.40). El Análisis Factorial Confirmatorio validó la estructura propuesta de ítems por dimensión.

**Conclusión.:**

La escala IME.Cat mostró valores psicométricos sólidos para evaluar competencias emprendedoras en estudiantes de enfermería en sus dimensiones, las cuales son fundamentales en el desarrollo profesional de la enfermería.

## Resumo

**Objetivo.:**

Desenvolver uma escala válida e confiável para medir as competências empreendedoras dos alunos de enfermagem, avaliando o nível de desenvolvimento em várias dimensões do empreendedorismo.

**Métodos.:**

O Instrumento de Medição de Empreendedorismo da Catalunha (IME.Cat) foi construído com a adaptação de dois instrumentos existentes, e um estudo psicométrico foi realizado para abordar a validade de conteúdo, a validade de construção e a confiabilidade. A consistência interna e a capacidade discriminatória dos itens do instrumento foram examinadas.

**Resultados.:**

A escala IME.cat apresentou alta confiabilidade (α=0.89) para o conjunto completo de 25 itens. Os valores de α de Cronbach para as dimensões individuais foram: Tratamento de problemas=0.78; Criatividade=0.76; Segurança pessoal=0.64; e Aceitação de riscos=0.46. Os índices de homogeneidade corrigidos para cada item do instrumento foram altos (>0.40). A análise fatorial confirmatória validou a estrutura de itens proposta por dimensão.

**Conclusões.:**

A escala IME.Cat apresentou bons valores psicométricos para avaliar as competências empreendedoras dos estudantes de enfermagem em suas dimensões, que são fundamentais para o desenvolvimento profissional da enfermagem.

## Introduction

In the last few years, a transformation has been observed towards new social customs. These social changes affect many health professions, such as nursing, with many internal changes observed in the nursing profession and the structures of health systems.^1^ The subject of entrepreneurship in nursing has been gaining importance in the last few years, and experts in the area of nursing regard entrepreneurship as a way to innovate and improve patient care, and to create business and employment opportunities for nursing. In general, the entrepreneurial character can be defined as the realization or introduction of something new and different, as opposed to what is traditionally done, based on the identification of opportunities and necessities.

Currently, nursing study plans tend to focus on the clinical and care aspects, and do not tend to integrate entrepreneurship training, despite the belief that the phenomenon of entrepreneurship must be addressed from the area of education.^2^ It has been observed that some countries already include this training. For example, Brazil, at the Federal University of Santa Catarina, teaches entrepreneurship in the initial training of nurses in an optional class in the second semester, named “Labor Market in Nursing and new modalities of service provision”. The methodology utilized idea workshops and collective discussions that arose after the visits to private social businesses in Florianópolis.^3^

At the university of Massachusetts (UMass), a proposal has been made for a comprehensive certification program of nursing innovation and entrepreneurship (INNOVAR), designed for remodeling bachelor’s education, by adopting a new vision of innovation in nursing and entrepreneurship education.^4^ In the remaining countries consulted, such as Canada, Australia, and the United Kingdom, entrepreneurship was relevant in the practice of professionals, and efforts were made towards professional planning. In Canada, in 1930, 60% of the nurses exerted their profession as entrepreneurs.^5^ The Royal College of Nursing Australia^6^ and the Nurses Association of New Brunswick^7^ delve into the characteristics of these professionals, exploring their activities, promoting good practices, and having an effect on training about leadership and the assumption of new roles in nursing. However, it was not observed that entrepreneurship is considered in their initial training for the development of projects related with the care of people’s lives and health. The entrepreneurship training is provided as continuous education.

In agreement with Camarillo *et al*.,^8^ nursing professionals lack academic training that allows them to see that after finishing university, they could establish businesses for caring for people’s health, by performing healthcare functions to promote health, prevent disease, or interventions to comply with a specific healing and/or rehabilitation treatment. The university study programs that offer nurse training do not include courses that let the future graduates visualize caring for health as an attractive option. In order for nurses to play a leading role in health innovation in the initial training of nurses, they must strategically think about the knowledge and skills that the next generation of nurses will need, and the support these needs.^9^ One of the key elements is to develop talent, the ability to adapt to fast and constant changes, to identify opportunities in the area of health, to become a fundamental element in the generation of ideas, and the creation of new products and services. 

Entrepreneurship education must be fostered to make all of these ingredients a reality.^10^ Entrepreneurship is a competence that encompasses a set of abilities and skills that are demanded in personal, professional, and social areas,^11^ such as: (i) Nursing management and leadership: developing the entrepreneurship initiative and spirit (ESIM) and the creative and entrepreneurship capacities (capacity to formulate, design, and manage projects/ capacity to seek and integrate new knowledge and attitudes); (ii) Innovation and entrepreneurship in nursing: demonstrate entrepreneurial initiative, motivation, and spirit with respect to self-learning and the professional activity (iii) Leadership in nursing: develop entrepreneurial initiative and spirit to create innovative and competitive proposals in the area of discipline; and: (iv) Management and innovation of nursing care: develop entrepreneurial initiative and spirit. 

Catalonia offers some innovative experiences in the university training of nurses. Thus, the Escuela Universitaria de Enfermeria Gimbernat (EUEG) of Barcelona offers the mention of Leadership, Innovation, and Emerging Roles in Nursing, where the students can enroll in the course Innovation and Entrepreneurship in Nursing, for a total of 8 ECTS (European Credit Transfer System). The aim of this course is to: i) Promote learning competences linked to creativity, innovation, and entrepreneurship. ii) Receive expanded learning within the nursing discipline, that is, at the same time, different from the current theoretical contents found in the nursing degree, which are exclusively based on health care and assistance.

This training seeks to focus on personal and professional characteristics, such as Independence, innovation, and responsibility, and to drive innovation in health care. In summary, the intention is for students to understand the process of introduction and management of change to determine and play an active role as an agent of change, starting with this initial training.^12^ Within the framework of this training, the question was raised whether there could be an entrepreneurship measurement scale to be used in nursing training, and to help with the analysis of the changes observed during the training, and the impact on the professional practice of the graduate after receiving training on Innovation and Entrepreneurship. The objective of the study was to develop a valid and reliable scale to measure entrepreneurship competences, by assessing the level of development of diverse entrepreneurship dimensions. This scale will allow us to better understand the usefulness and applicability of the entrepreneurship competences acquired in the training, and their transfer to the area of healthcare where they exert their profession. 

## Methods

To answer the objective of the study, an instrument was created starting with the Measurement Instrument of Entrepreneurship Attributes (IMAE).^12^ Some of the sub-competencies of this instrument were posteriorly adapted, resulting in the Questionnaire of Competences in University Entrepreneurship (CCEU).^13^The first instrument by Alcaraz,^12^ contained 99 items and measured factors that the author considered as key factors, which included 6 competences for entrepreneurship: risk tolerance, recover and learn from mistakes, identify opportunities, propose innovative solutions, obtain resources, and implement innovative solutions. 

The second instrument, CCEU, is an instrument adapted from diverse validated questionnaires, and within it, different attributes are chosen that identify entrepreneurship competences in university students. It is composed of 25 items where the most notable features of the entrepreneur profile are considered, and where sub-competencies from each of them are chosen, with a series of entrepreneurship attributes that are compiled in 4 groups: Identify opportunities, develop solutions, learn from failure, and awareness of entrepreneurship. This instrument brings the possibility that the students become aware of their areas of opportunity in the sub-competences assessed. Starting with both instruments, the new instrument was constructed, taking into account the following aspects: (i) Identify equivalencies between the dimensions in both instruments of reference, and combine them into the new proposal; (ii) Identify the items corresponding to each dimension in the reference instruments as equivalent, eliminating those that are more redundant, to construct the proposal in a more reduced manner; (iii) Study the items that better fit with the Catalonian reality, as the resulting instrument would later be translated to Catalan to be applied in this context; (iv) Include those items that specifically express the Catalonian reality; and (v) Verify that all the dimensions and items fit within the objectives defined in the study. A traceability table was created.

At the end of the adaptation process of the IMAE and CCEU, the Entrepreneurship Measurement Instrument Catalonia (IME.Cat) was obtained. This instrument is composed of 24 items with a Likert scale format with 4 response options: “Definitely doesn't describe me” = 1; “Probably doesn't describe me” = 2; “It probably does describe me” = 3 and, “It definitely does describe me” = 4. It is organized around 4 dimensions: (1) *Problem management*: ability to identify discrepancies between an actual state and desired one, and posteriorly, act to resolve the discrepancy, with 9 items; (2) *Risk Acceptance:* willingness to face risks in search of better results, with 3 items; (3) *Creativity*: thinking ability to generate new, original, and valuable responses, with 8 items; and (4) *Personal confidence* (Self-confidence and Perseverance), with 5 items.

After the construction of the scale, it was subjected to content validity through experts’ judgement. Eight experts were selected independently according to the following criteria: experience on the subject, diversity of perspectives, ability to provide constructive feedback, independence, and objectivity, informed consent to participate in the process, and availability. Following these criteria, two different groups composed of 4 participants from the sample, and 4 experts selected due to their experience in the area of nurse entrepreneurship were created. The experts had to assess the criteria of univocity, suitability, and relevance, as shown in Table 1.

**Table 1:** Validation criteria

Criteria	Definition	Score		
Univocity	If the item has only one meaning or is only use with a single and unique meaning	Si	1
		No	0
Suitability	If the questions are considered adequate according to the information that the instrument intends to collect	Si	1
		No	0
Relevance	The item is appropriate for representing the content of the construct of the variable under study	Si	1
No	0

The critical value from which the items were analyzed was 80%, reformulating the items that did not reach this value.

The reliability of the scale and its parts was analyzed. To ensure the quality criteria demanded from assessment instruments, the content and construct validity and the reliability were analyzed, by studying the internal consistency and discrimination capacity of the items of the IME.Cat instrument. The reliability of set of items was analyzed by calculating the Cronbach’s “alpha” reliability coefficient, which addresses this property of the instrument from a perspective of internal consistency. This coefficient was calculated with a 95% confidence, for the complete scale and for each of the 4 dimensions. For the analysis of the structural validity of the items, a Confirmatory Factor Analysis (CFA) was performed, to verify if the structure proposed, with the previously-known four dimensions, was replicated in our study, at least in an acceptable manner.

*Sample.* The scale was applied to a sample of 230 participants, who were selected randomly, by convenience, and who had previously signed an informed consent, which underlined the voluntary participation in the study and indicated that the personal data would be protected and treated with absolute confidentiality in accordance to the current laws on the treatment and protection of data. Thus, the sample was composed of nursing students and graduates who were enrolled or had taken the Nursing Innovation and Entrepreneurship course, within the training framework of the Escuela Universitaria de Enfermería Gimbernat-Barcelona (EUEG). The significant characteristics of the sample indicated a clear majority of women as compared to men (83.5% vs 16.5%). Their age ranged from 21 to 46 years old, with a mean of 25 and a standard deviation of ±5.0 years. The distribution showed a clear tendency towards younger participants, and also indicating that the mean age of women was somewhat lower than the mean age of the men (24.8 vs 28.0 years old).

Ethical considerations of the study. The study followed the following ethical principles: (i) Informed consent from the participants before gathering any data, as well as their voluntary declaration to participate; (ii) Minimization of data, only collecting the minimum amount of data necessary to meet the research objectives. Personal and sensitive data were not collected, only the basic data that provided sociodemographic information; (iii) Anonymization and pseudonymization of the data with codes, so that the data could not be directly linked; (iv) Safety of the data and storage for a pre-determined amount of time; (v) Transparency: the participants were provided information about the objective of the study, the protection of data, the possibility of correcting the incorrect data, or remove their consent at any point in time; and (vi) Compliance with the data protection laws and any ethical regulations related with research. Work was not performed with sensitive data, and for this reason, it was accepted by the Doctoral School of the University of Lleida.

## Results

### Validation through judges

The judges pointed to the need to make adjustments to items 16, 18, 22, and 23. They were re-written, and a linguistic correction was performed of the items, according to their indications. The final result was the IME.Cat, composed of 25 items framed within 4 Dimensions: Problem management, Risk acceptance, Creativity, and Personal confidence.

### Reliability

The results showed that the reliability of the entire set of 25 items was high (0.89), which guarantees the trustworthiness of the answers provided by the sample of participants. Next, a reliability study was performed according to the dimensions. The results showed that the reliability was good in three of the four dimensions (0.64; 0.76 and 0 .78). The coefficient was poor (0.43) only in the dimension Risk acceptance, which could be associated to the low number of items in this dimension (3). Thus, the number of items in this dimension may have to be increased to improve its reliability. The results from the reliability analysis of the instrument according to the dimensions are summarized in Table 2 according the different dimensions:

**Table 2:** Reliability analysis

Dimensions	Number of items	Cronbach’s Alpha	95% CI
Problem management	9	0.78	0.70 a 0.84
Risk acceptance	3	0.43	0.12 a 0.65
Creativity	8	0.76	0.67 a 0.83
Personal confidence	5	0.64	0.50 a 0.75
Complete questionnaire	25	0.89	0.86 a 0.92

The corrected homogeneity indices (CHI) of each of the items of the instrument are shown in Table 3. As shown, most obtained high values (>0.40), which strongly contribute to the reliability of the whole instrument. However, an exception was found in an item that was somewhat weaker, item 4, whose CHI was found at the threshold (0.20), which indicates that it should be eliminated from the questionnaire. In fact, its elimination would notably improve the reliability of the dimension to which it belongs (Risk acceptance, the one with the worse reliability), from 0.43 to 0.54, although its effect on the reliability of the whole instrument is minimal (from 0.89 to 0.90). This low effect resulted in the maintenance of item 4, because, for the research study, it meant obtaining data of interest on the participant’s ability to assume risks.

The results obtained allow us to accept that the reliability of the measurement instrument is sufficiently guaranteed. Item 4 was not eliminated, as it would not greatly affect the whole instrument, and given the objective of the study, it was believed that it was a good example to better understand risk assumption.

**Table 3:** Reliability according to item and correlation coefficients item-dimension of the Entrepreneurship Measurement Instrument Catalonia (IME.Cat) (*n*=230).

Dimension and items	Reliability				item-dimension correlation
	Dimension		Questionnaire		
	CHI	REI	CHI	REI	
*Problem management (0.78)*					
Item 2: I am sometimes wrong and I make mistakes, but I know I can do things right	0.35	0.77	0.36	0.89	0.47
Item 5: When I want something, I insist until I get it	0.55	0.74	0.60	0.89	0.72
Item: I consider myself a resourceful person, especially when difficult situations arise	0.54	0.75	0.62	0.89	0.70
Item 16: I am excited to do new and unusual things	0.34	0.77	0.37	0.89	0.45
Item 17: I believe that in life you have to take risks to earn more or achieve higher goals	0.31	0.78	0.41	0.89	0.42
Item: I often find quick and effective solutions to problems.	0.60	0.74	0.63	0.89	0.75
Item 21: When I think about starting a business, I'm not afraid of the idea of the unknown	0.50	0.75	0.53	0.89	0.64
Item 24: I don't get discouraged if I encounter obstacles to achieving my goals	0.41	0.77	0.43	0.89	0.56
Item 25: Even if I don't achieve my goals, I don't lose interest in what I do	0.56	0.74	0.58	0.89	0.68
*Risk acceptance (0.43)*					
Item 4: When I invest my money I prefer to risk it on something that can give me more profits than on a fixed-term deposit	0.20	0.54	0.23	0.90	0.40
Item 6: I can solve problems quickly, even under pressure	0.30	0.31	0.52	0.89	0.74
Item 15: I am confident in my own ideas and possibilities	0.37	0.19	0.63	0.89	0.79
*Creativity (0.76)*					
Item 1: I often have original ideas and put them into practice.	0.44	0.73	0.40	0.89	0.61
Item 7: I see creative possibilities (of innovation) in everything I do	0.52	0.72	0.47	0.89	0.70
Item 11: I enjoy looking for new ways of seeing things, instead of being guided by already known ideas	0.59	0.71	0.58	0.89	0.74
Item 12: I find risk exhilarating	0.36	0.75	0.43	0.89	0.48
Item 14: I believe in the saying: He who does not risk does not gain	0.28	0.76	0.35	0.89	0.40
Item 19: I am good at facing a large number of problems at the same time	0.54	0.71	0.56	0.89	0.70
Item 22: I feel safe even when someone criticizes what I have done	0.47	0.73	0.59	0.89	0.61
Item 23: When I face a problem, I like to find new ways to solve it	0.43	0.74	0.44	0.89	0.60
*Personal confidence (0.64)*					
Item 3: When I want something, I insist until I get it	0.41	0.58	0.46	0.89	0.66
Item 8: I firmly believe that I will be successful in everything I set out to do	0.53	0.51	0.50	0.89	0.76
Item 9: I firmly believe that if I don't succeed the first time, I should try again and again	0.41	0.58	0.46	0.89	0.66
Item 13: I am convinced of my capabilities and I know very well how to exploit them	0.38	0.60	0.48	0.89	0.62
Item 20: I believe that perseverance (consistency) is important to achieve success	0.26	0.64	0.28	0.89	0.48

CHI = Corrected item reliability; REI. = Reliability excluding the item

### Validity of the instrument

The sample size was very low (only *n*=230) for the mathematical requirements of a CFA, so that the fit indices that assess the suitability of the use of this procedure with our data provided results that indicated a lack of fit (RMSEA>0.08). However, it contains the values of the correlation coefficients item-dimension and dimension-dimension. These coefficients allow us to admit that each of the items belongs to its expected dimension, as well as the correlations between dimensions. Thus, according to the data shown in Table 3, a good correlation can be observed between all the dimensions, especially between Problem management and Personal confidence (0.60), aside from Risk acceptance with Personal confidence (0.62). The lowest values were those that related the dimension Creativity with the dimensions Problem management (0.33) and Personal confidence (0.40); nevertheless, as a function of all these data, the structure proposed is deemed sufficiently proven.

**Table 3:** Correlation coefficients between the IME.Cat dimensions

Dimensions	PM	RA	C	PC
Problem Management (PM)	1.00	0.55	0.33	0.60
Risk acceptance (RA)		1.00	0.50	0.62
Creativity (C)			1.00	0.40
Personal Confidence (PC)				1.00

## Discussion

The results from the present study demonstrate that the IME.Cat scale is a good tool for measuring the inherent skills in the entrepreneurship competence, and more specifically, those that shape the different dimensions of the IME.Cat: Problem management, Risk acceptance, Creativity, and Personal confidence (Self-confidence and Perseverance). It is important to have a scale of these characteristics, as the entrepreneurial culture gives confidence to people when promoting new ideas that allow students to create, execute, and perform any project they may want to pursue, hence the importance of the entrepreneurship course in personal and professional development.^13^ Entrepreneurs must be aware of new opportunities.^14^ Some of the personal characteristics form a synergy, such as the knowledge acquired, personal skills, experience, emotional intelligence, and the values that identify the abilities of a person to become an entrepreneur.^15^

In the modern context, entrepreneurship in Nursing is important for widening the visibility and consolidation of the profession as a science, a technology, and an innovation, in the most diverse scenarios and fields of action. Only then will society become aware of the advances in the profession, through its social mission, and its achievements in health. Addressing the concept of entrepreneurship, then, guides the promotion of visibility of nursing, as well as the achievements of new levels of professional development for nurses. ^16^

It is important to train nursing professionals in entrepreneurial competencies, and the IME.Cat scale tool will allow making a diagnosis before the training intervention, performing an analysis of the progress achieved and the consolidation of the skills that were worked on. Education in entrepreneurial competencies will help us understand nursing in the 21st Century, as Leray, Villarruel and Ritchmond^17^ proposed, with respect to strategic thinking, knowledge, and the skills that will be needed by the next generation of nursing professionals. Among the systematic competences found in the Tuning project,^18^ we highlight the ability to adapt to new situations, the ability to create new ideas, creativity, leadership, the design and management of projects, entrepreneurship initiative and spirit, and the motivation of achievement. 

Although this instrument was applied and validated with a sample composed of nursing students, it would be equally applicable to any area of training, as it proposes items and dimensions that define entrepreneurship, and the IME.Cat emerged from other previous versions with generic characteristics. However, in this case, the focus was to deal with training challenges of nursing healthcare personnel, in which a fundamental task is to provide meaning to entrepreneurship, by developing competences in that field.^19^ Therefore, the IME.Cat scale is a valid and decisive instrument in the challenge of professional development of nurses. The results obtained in the dimension Risk acceptance, and more specifically, in item 4, the Confirmatory Factor Analysis and the reliability analysis made us believe that more items should be added to this dimension, before deciding to eliminate item 4. This section is important in the training of nurses, as it is an essential skill that not only contributes towards their professional development, but also promotes the ability to continuously adapt, innovate, and improve healthcare. It is a key element for preparing future nurses to be able to face the dynamic and changing challenges in the area of patient care.^20^

In conclusion, the study highlights the efficiency of the IME.Cat scale as a valuable tool for measuring the values inherent to the entrepreneurial competency, especially in key dimensions such as Problem management, Risk acceptance, Creativity, and Personal confidence. Entrepreneurship in nursing in presented as essential in the modern context, to broaden the visibility and to consolidate the profession in diverse scenarios. Training in entrepreneurial competencies is supported by the IME.Cat scale, which is positioned as a tool that can be used to diagnose and analyze the progress in the development of entrepreneurial competences in nursing professionals. Without a doubt, the entrepreneurial competency allows nurses to create value when recognizing and taking advantage of opportunities, making decisions with limited information, and staying adaptable and resilient against conditions that are frequently uncertain and complex.

*Limitations of the study.* We believe that the IME.Cat scale is valid and reliable. However, the present study has two weaknesses: firstly, the dimension Risk acceptance would benefit from an improvement in its reliability by increasing the number of items that define it. In second place, this scale was applied to students in a specific center, and this could bias the results obtained. It would be interesting to broaden the sample to other contexts. Nevertheless, this does not invalidate the results obtained.
